# Rapid and Sustained Response of Biochemically Uncontrolled Acromegaly to Once-daily Oral Paltusotine Treatment

**DOI:** 10.1210/clinem/dgaf579

**Published:** 2025-10-23

**Authors:** Beverly M K Biller, Alessandra Casagrande, Atanaska Elenkova, Cesar L Boguszewski, Raquel S Jallad, Beibei Hu, Erika Hubina, Pouneh K Fazeli, Maria Fleseriu, Peter J Snyder, Christian J Strasburger, Martin Bidlingmaier, Yining Zhao, Beatriz Soares, Peter J Trainer, R Scott Struthers, Alan Krasner, Mônica R Gadelha

**Affiliations:** Neuroendocrine and Pituitary Tumor Clinical Center, Massachusetts General Hospital, Boston, MA 02114, USA; Crinetics Pharmaceuticals, Inc, San Diego, CA 92121, USA; Department of Endocrinology, Medical University-Sofia, USHATE “Acad. Ivan Penchev”, Sofia 1431, Bulgaria; Department of Internal Medicine, Endocrine Division (SEMPR), Federal University of Paraná, Curitiba 80030-110, Brazil; Neuroendocrine Unit, Division of Endocrinology and Metabolism, Clinics Hospital, University of São Paulo Medical School, São Paulo 01246-903, Brazil; Crinetics Pharmaceuticals, Inc, San Diego, CA 92121, USA; 2nd Department of Internal Medicine, Division of Endocrinology, Central Hospital of Northern Pest—Military Hospital, Budapest 1134, Hungary; Neuroendocrinology Unit, Division of Endocrinology and Metabolism, University of Pittsburgh School of Medicine, Pittsburgh, PA 15213, USA; Pituitary Center, Departments of Medicine and Neurological Surgery, Oregon Health and Science University, Portland, OR 97239, USA; Division of Endocrinology, Diabetes and Metabolism, Department of Medicine, University of Pennsylvania, Philadelphia, PA 19104, USA; Department of Medicine for Endocrinology and Metabolism, Charité Universitaetsmedizin, Campus Mitte, Berlin 10117, Germany; Medizinische Klinik und Poliklinik IV, LMU Klinikum, Munich 80336, Germany; Department of Neurosurgery, University Hospital Erlangen, Erlangen 91054, Germany; Department of Internal Medicine, Medical School of the Federal University of Minas Gerais, Belo Horizonte 31270-901, Brazil; Crinetics Pharmaceuticals, Inc, San Diego, CA 92121, USA; Crinetics Pharmaceuticals, Inc, San Diego, CA 92121, USA; Crinetics Pharmaceuticals, Inc, San Diego, CA 92121, USA; Neuroendocrinology Research Center/Endocrinology Division, Medical School and Hospital Universitario Clementino Fraga Filho, Universidade Federal do Rio de Janeiro, Rio de Janeiro 21941-913, Brazil

**Keywords:** acromegaly, paltusotine, IGF-I, somatostatin, somatostatin receptor 2, clinical trial

## Abstract

**Context:**

Paltusotine is a nonpeptide, selective somatostatin receptor 2 agonist in development as once-daily oral treatment for acromegaly.

**Objective:**

To evaluate efficacy and safety of paltusotine in patients with biochemically uncontrolled acromegaly not currently receiving medical therapy.

**Methods:**

In this phase 3, randomized, double-blind, placebo-controlled trial, adults with medically untreated acromegaly at randomization (stratum 1: medication-naïve or off acromegaly medications ≥4 months [IGF-I ≥ 1.3 × upper limit of normal {ULN}]; stratum 2: controlled on a somatostatin receptor ligand and underwent washout [IGF-I increase ≥30% to ≥1.1 × ULN]) received paltusotine or placebo for 24 weeks.

**Results:**

A total of 111 patients (stratum 1, n = 82; stratum 2, n = 29) enrolled (paltusotine, n = 54; placebo, n = 57). The primary endpoint of IGF-I normalization at 24 weeks was met in 55.6% of paltusotine-treated patients vs 5.3% for placebo (odds ratio [OR]: 42.81; 95% CI, 8.44-455.82; *P* < .0001), with superiority to placebo in both strata. Paltusotine treatment decreased IGF-I in 92.6% of patients within the first 4 weeks. All secondary endpoints were met: mean (±SE) change in IGF-I of -0.82 ± 0.08×ULN with paltusotine vs 0.09 ± 0.08×ULN with placebo (*P* < .0001); IGF-I < 1.3×ULN in 66.7% vs 14.0% of patients (OR: 18.32; 95% CI, 5.64-79.16; *P* < .0001); GH (5-sample mean) < 1.0 ng/mL in 57.4% vs 17.5% (OR: 7.59; 95% CI, 2.78-23.48; *P* < .0001); mean (±SE) change in Acromegaly Symptom Diary score of -2.7 ± 1.4 vs 2.8 ± 1.4 (*P* = .004). Most adverse events were acromegaly symptoms or mild, transitory gastrointestinal effects characteristic of somatostatin receptor ligands (eg, diarrhea, abdominal pain). Pituitary tumor volume was stable or reduced in paltusotine-treated patients.

**Conclusion:**

IGF-I normalized in significantly more patients with uncontrolled acromegaly treated with paltusotine vs placebo. Paltusotine was associated with rapid, sustained IGF-I reduction, significant symptom improvement and stable or reduced pituitary tumor size and was well tolerated.

The current first-line medical option for most patients with acromegaly is a monthly depot injection of either of the peptide somatostatin receptor ligands (SRLs) octreotide or lanreotide (1, 2). These peptide analogs act predominantly via the somatostatin receptor 2 (SST2) to inhibit GH secretion and, consequently, reduce IGF-I production (2-5). While the availability of SRLs has greatly improved the treatment of acromegaly, depot injections are associated with limited treatment satisfaction (6), substantial patient burdens such as injection site pain and reactions, prolonged dose titration periods (3 months until steady-state drug exposure is achieved per dose strength) (7), and repeat office visits for administration by trained health care professionals (8, 9). Other complications of SRL injections include suboptimal accuracy of drug delivery and the formation of subcutaneous nodules (9-13). Moreover, variable symptom control is frequently reported with SRL injections, described as differences in symptom control from month to month or as symptom worsening before the next injection (8, 9). Consistent with the latter pattern is the finding that a subset of patients who are thought to be “well controlled” using injected SRLs can experience elevations in IGF-I in the final portion of the injection cycle (14). To date, the only approved oral medication in this class is enteric-coated octreotide capsules, which is indicated for patients who previously responded to an injected depot SRL (1, 2).

Paltusotine is a nonpeptide, highly selective SST2 receptor biased agonist designed using the techniques of iterative medicinal chemistry to address many of the shortcomings of first-line medical therapies for acromegaly (15). Paltusotine is >4000-fold selective for the SST2 receptor relative to other SST receptor subtypes and is biased to activate Gi signaling preferentially, relative to internalization/desensitization pathways (15, 16). Paltusotine is efficiently absorbed through the gastrointestinal tract, allowing for oral administration; rapidly reaches steady-state drug exposure (within 1 week); and has a long circulating half-life of approximately 30 hours, suitable for once-daily dosing (17). A phase 1 study in healthy volunteers showed that GH releasing hormone-stimulated GH levels were reduced after a single dose of paltusotine and IGF-I levels were reduced within 10 days of daily dosing (17). An open-label, single-arm, phase 2 study indicated that paltusotine could provide maintenance of suppressed IGF-I levels in patients with acromegaly who were switching from depot SRL injections (18).

The first phase 3, randomized, placebo-controlled trial of paltusotine in acromegaly (PATHFNDR-1; NCT04837040) demonstrated that both biochemical and symptom control were maintained in patients who switched from monthly injected SRLs to once-daily paltusotine (19). Here, we present results from a second phase 3, randomized, placebo-controlled trial (PATHFNDR-2) to evaluate the safety and efficacy of paltusotine in patients with acromegaly who were not receiving medical therapy and were biochemically uncontrolled.

## Materials and Methods

### Study Design

**Figure 1.**
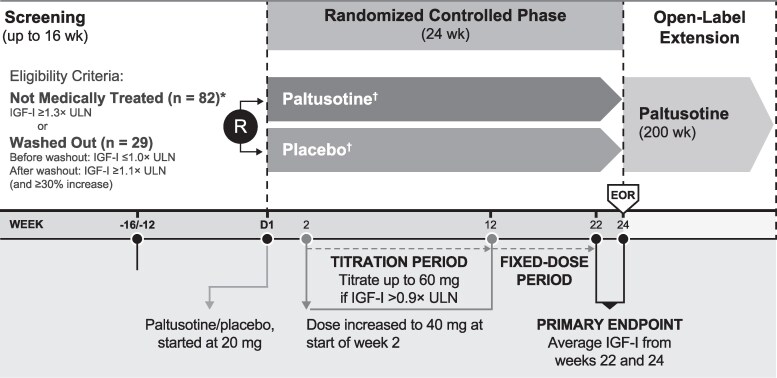
Study design. *Medication-naïve, n = 46; previously treated (off acromegaly medications for ≥4 months), n = 36. ^†^Patients received rescue medication (injected depot somatostatin receptor ligand) if the following criteria were met: 2 consecutive IGF-I levels ≥1.5 × ULN at the highest dose of study medication (60 mg/day) and exacerbation of acromegaly symptoms for ≥2 weeks, as assessed by the investigator.

PATHFNDR-2 (Paltusotine Acromegaly Therapy Featuring a Non-invasive Daily Regimen; NCT05192382) was a multinational, phase 3, randomized, double-blind, placebo-controlled trial conducted at 57 centers in 15 countries. The study included a screening period, a 24-week randomized controlled phase, and an open-label extension (OLE) phase that is currently ongoing (Fig. 1).

The study was conducted in accordance with the Guideline for Good Clinical Practice of the International Conference on Harmonization, the ethical principles of the Declaration of Helsinki, and local regulatory guidelines. An institutional review board or independent ethics committee at each site approved the protocol, and all patients provided written informed consent.

### Patients

Patients were adults (≥18 years of age) with a confirmed diagnosis of acromegaly that was biochemically uncontrolled at the time of randomization. Based on acromegaly treatment history, patients were stratified, as prespecified in the protocol, as medically untreated (stratum 1) or washed out (stratum 2). Stratum 1 included patients who were medically untreated and had IGF-I ≥ 1.3 times the upper limit of normal (× ULN) at screening (either medication-naïve patients with at least 1 pituitary surgery at least 3 months previously or patients off acromegaly medications for at least 4 months before screening). Stratum 2 included patients controlled (IGF-I ≤ 1.0 × ULN) on octreotide or lanreotide who underwent washout, after which IGF-I levels increased by at least 30% and to a value of ≥1.1 × ULN. Patients were excluded from the study if they had been nonresponsive to or unable to tolerate previous treatment with octreotide or lanreotide (as determined by the investigator), had received pituitary radiation therapy within 3 years of screening, or had poorly controlled diabetes (hemoglobin A1c ≥ 8.5%) or cardiovascular, renal, or hepatic disease. Patients were also excluded for use of long-acting pasireotide (within 24 weeks of screening), pegvisomant or dopamine agonists (within 16 weeks), any combination of 2 or more acromegaly medications, or proton pump inhibitors. Oral estrogen was permitted if use began at least 12 weeks before screening.

### Randomization

Eligible patients were randomly assigned (1:1) to paltusotine or placebo using an interactive web response system. Randomization was stratified based on prior acromegaly treatment (stratum 1 or stratum 2). All investigators, trial personnel, and patients were unaware of the treatment assignments.

### Interventions

Study medication consisted of paltusotine tablets or matching placebo. Patients were instructed to take study medication with water once daily in the morning, after an overnight fast of at least 6 hours, and then to wait an hour before eating or drinking anything other than water. The starting dose of paltusotine was 20 mg/day, which was increased to 40 mg/day at week 2, provided tolerability was acceptable. The dose was further uptitrated to 60 mg/day during weeks 6 through 12 if IGF-I was >0.9 × ULN. The protocol specified no dose uptitration during the fixed-dose phase of the study (final 12 weeks of treatment period); down-titration (to as low as 20 mg/day) was allowed at any time based on tolerability.

Per protocol, injected octreotide or lanreotide was administered if both of the following rescue criteria were met: 2 consecutive IGF-I levels ≥1.5 × ULN at the highest dose of study medication (60 mg/day) and exacerbation of acromegaly symptoms for ≥2 weeks as assessed by the investigator.

### Study Endpoints and Assessments

The primary endpoint was the proportion of patients achieving biochemical normalization (IGF-I ≤ 1.0 × ULN) at the end of the randomized controlled phase (mean of weeks 22 and 24). Patients who received octreotide or lanreotide rescue were designated as having not achieved biochemical normalization. The key secondary endpoint was the change from baseline in IGF-I (expressed as ×ULN) at the end of randomized treatment (mean of weeks 22 and 24 if no rescue medication or the last assessment before rescue if rescued). Other secondary efficacy endpoints were the proportion of patients with IGF-I < 1.3 × ULN at the end of the randomized controlled phase (mean of weeks 22 and 24), the proportion of patients with mean 5-sample GH <1.0 ng/mL at week 22, and change from baseline in total Acromegaly Symptom Diary (ASD) score at the end of randomized treatment. Exploratory endpoints included the change from baseline in GH level at week 22 and the change from baseline in pituitary tumor volume at the end of the randomized controlled phase.

Serum IGF-I and GH were measured centrally using iSYS immunoassays (Immunodiagnostic Systems; Tyne & Wear, UK) and National Institute for Biological Standards and Control calibration standard 02/254 for IGF-I (20) and 98/574 for GH (21). Baseline IGF-I was calculated as the mean of the day 1 value and the last measurement before this day. Because of the pulsatile nature of GH secretion, the mean value was determined from 5 fasting samples collected at least 30 minutes apart within a 3-hour period.

The ASD is a daily patient-reported outcome measure of acromegaly symptom severity that was developed in alignment with US Food and Drug Administration guidance for use in evaluating the symptom experience of patients with acromegaly in clinical trials (22). The ASD includes 7 core acromegaly symptoms: headache, joint pain, sweating, fatigue, leg weakness, swelling, and numbness/tingling (22). Patients rated these symptoms for the previous 24 hours on a scale from 0 to 10, with higher scores indicating worse symptom severity. The ASD total score was the sum of scores for the 7 core symptoms (range, 0-70).

Pituitary tumor volume was measured at baseline and at the end of the randomized treatment period (week 24) from magnetic resonance imaging (MRI) scans performed locally in accordance with specified image acquisition standards. Scans were evaluated centrally by 2 experienced readers who were blinded to treatment assignment. The image analysis procedure has been described previously (19).

Safety assessments included adverse event (AE) monitoring, clinical laboratory tests, vital signs, electrocardiogram, and biliary/gallbladder ultrasonography (at screening and week 24). For each reported AE, the investigator was asked to assess whether it was considered a symptom of the patient's acromegaly or related to study medication. Treatment adherence was assessed at each study visit by direct questioning and/or tablet counts of returned study medication.

### Statistical Analysis

Assuming an equal number of patients in each stratum and a discontinuation rate of 10%, a sample size of 76 patients (38 per treatment group) was originally planned to provide 90% power for a logistic regression analysis of paltusotine vs placebo with 2-sided alpha = .05. Because of greater enrollment in stratum 1 (either medication-naïve patients or patients off acromegaly medications for at least 4 months before screening) vs stratum 2 (washed out from injected SRL) at the time that 75% of patients were randomized, a predefined sample size reestimation was applied, which allowed enrollment to continue to 111 patients.

The proportion of patients who met the primary endpoint was evaluated using an exact logistic regression model with treatment group as a factor and stratum as a covariate. Patients were considered to have met the primary endpoint if IGF-I was ≤1.0 × ULN based on the average of weeks 22 and 24. If only 1 measurement was available (week 22 or 24), it was used to evaluate the primary endpoint. If IGF-I values were missing at both weeks 22 and 24 or if study treatment was discontinued for any reason (including meeting protocol criteria for rescue therapy), patients were considered to have failed to meet the primary endpoint.

A fixed sequential testing procedure was used to control the family-wise type I error rate. If the primary analysis was significant (predefined alpha level, .05), secondary endpoints were analyzed in this order: change from baseline in IGF-I, proportion of patients with IGF-I < 1.3 × ULN, change from baseline in ASD score, and proportion of patients with 5-sample mean GH <1.0 ng/mL at week 22. Change in IGF-I and change in total ASD score were analyzed using worst-rank score analysis of covariance, with treatment group as a factor and stratum as a covariate. Change in each individual ASD item score was evaluated in a post hoc analysis of covariance, with treatment group and stratum as fixed effects and baseline value as a covariate. The proportion of patients who met the specified IGF-I or GH thresholds was analyzed using the same methodology as for the primary endpoint.

## Results

### Patients

**Figure 2.**
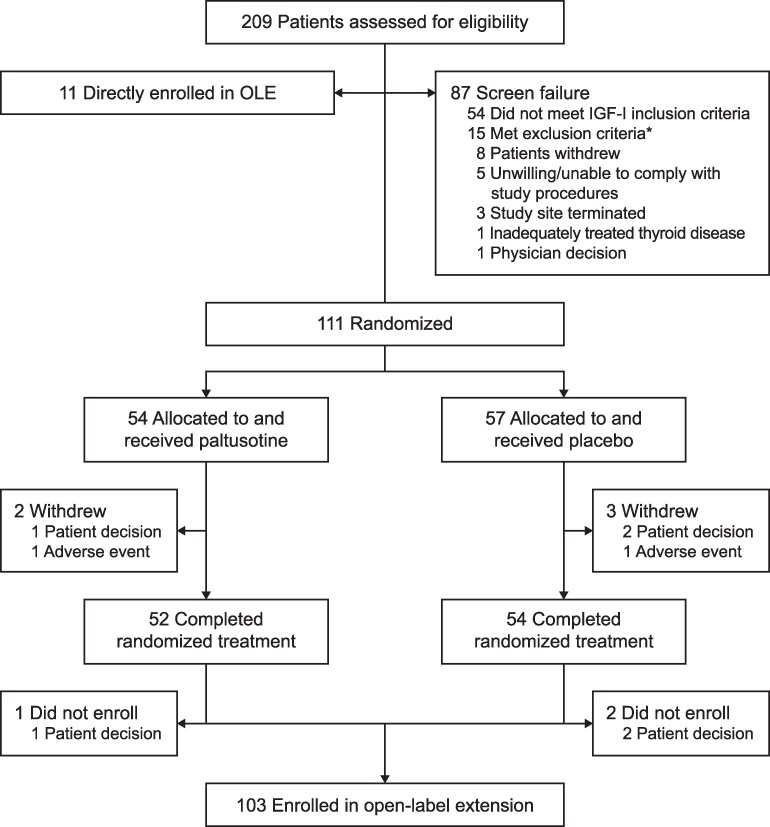
Patient disposition. *Inadequately controlled diabetes, clinically significant concomitant disease, active malignant disease within the past 5 years, history of hepatitis B or HIV, QT interval prolonged, elevated liver enzymes, high-risk pituitary tumor, symptomatic cholelithiasis, pregnant/lactating female, use of exclusionary medications.

The study was conducted from August 2022 through January 2024 (last visit in the randomized controlled phase). A total of 111 patients were randomized and received at least 1 dose of study medication (paltusotine, n = 54; placebo, n = 57). Of 106 patients who completed the randomized controlled phase, 103 (97.2%) enrolled in the OLE (Fig. 2). Treatment groups were balanced for demographics and disease characteristics, except for time since pituitary surgery being greater in the paltusotine group (Table 1). Mean baseline IGF-I concentration was comparable between the paltusotine (2.0 × ULN) and placebo (2.2 × ULN) groups. Mean baseline GH was greater in the placebo group because of 1 outlier (5-sample GH = 168.0 ng/mL; IGF-I = 2.3 × ULN), but median GH values were similar between groups (paltusotine, 2.1 ng/mL; placebo, 2.3 ng/mL). The mean rate of medication adherence was 97.7% and 99.6% for paltusotine and placebo, respectively. In the paltusotine group, the final dose at the end of the randomized treatment period was 60 mg/day in 66.7% (36/54) of patients, 40 mg/day in 27.8% (15/54), and 20 mg/day in 5.6% (3/54).

**Table 1.** Demographics and baseline characteristics by randomized treatment group

	Paltusotine (n = 54)	Placebo (n = 57)
Age, y, mean (SD)	47.5 (13.6)	45.9 (12.3)
Sex, n (%)		
Female	26 (48.1)	33 (57.9)
Male	28 (51.9)	24 (42.1)
Race, n (%)		
White	28 (51.9)	30 (52.6)
Asian	15 (27.8)	19 (33.3)
Black or African American	2 (3.7)	1 (1.8)
Multiple/other	6 (11.1)	4 (7.0)
Unknown	3 (5.6)	3 (5.3)
Duration of acromegaly (since diagnosis), n (%)		
<1 y	8 (14.8)	8 (14.0)
≥1 to <5 y	17 (31.5)	22 (38.6)
≥5 y	29 (53.7)	27 (47.4)
Previous pituitary surgery, n (%)*^a^*	50 (92.6)	49 (86.0)
Time since surgery, months, median (range)	66.5 (4-299)	22.0 (3-309)
Prior pituitary radiation, n (%)	2 (3.7)	3 (5.3)
Baseline IGF-I, ×ULN, mean (SD)	2.0 (0.8)	2.2 (1.1)
Baseline GH, ng/mL, mean (SD), median*^b^*	3.0 (2.9), 2.1	9.4 (24.1), 2.3
Baseline ASD total score, mean (SD)	17.5 (16.3)	15.5 (13.4)
Stratification (based on treatment history), n (%)		
Stratum 1 (medically untreated at screening, IGF-I ≥ 1.3 × ULN)	40 (74.1)	42 (73.7)
Medication-naïve	22 (40.7)	24 (42.1)
Previously treated (off medications for ≥4 months)	18 (33.3)	18 (31.6)
Stratum 2 (washed out, IGF-I increase ≥30% to ≥1.1 × ULN)	14 (25.9)	15 (26.3)

Abbreviations: ASD, Acromegaly Symptom Diary; ULN, upper limit of normal.

^*a*^Six patients had no history of pituitary surgery, and this information was missing for 6 patients.

^*b*^Mean from 5 samples collected at least 30 minutes apart over a 3-hour period during screening.

Patients were stratified for efficacy analysis based on acromegaly treatment history. Stratum 1 (n = 82) included 46 medication-naïve patients (paltusotine, n = 22; placebo, n = 24) and 36 previously treated patients (paltusotine, n = 18; placebo, n = 18) who had been off prior acromegaly medications for ≥4 months. Stratum 2 included 29 patients (paltusotine, n = 14; placebo, n = 15) washed out from SRL therapy. Prior acromegaly medications are summarized in Table 2. Reasons for stopping acromegaly therapy in the 36 previously treated patients were lack of insurance/economic reasons (n = 13), patient choice to stop treatment (n = 8), physician recommended drug holiday (n = 5), pituitary surgery (n = 3), and other reasons (n = 7). In this group, there were 13 patients (paltusotine, n = 7; placebo, n = 6) who had not achieved normal IGF-I when last observed on prior treatment, 14 patients (paltusotine, n = 8; placebo, n = 6) with normal IGF-I when last observed, and 9 patients (paltusotine, n = 3; placebo, n = 6) with unknown prior control.

**Table 2.** Previous acromegaly medications

	Paltusotine (n = 54)	Placebo (n = 57)
Medication-naïve (no prior acromegaly medication), n	**22**	**24**
Previously treated*^a^* (off medications for ≥4 months), n	**18**	**18**
Octreotide LAR	7	10
Lanreotide depot	5	4
Cabergoline	3	2
Pasireotide	1	1
Pegvisomant	1	0
Bromocriptine	1	1
Washed out, n	**14**	**15**
Octreotide LAR	6	11
Low dose (10 mg/month)	0	1
Mid dose (20 mg/month)	3	4
High dose (30 or 40 mg/month)	3	6
Lanreotide depot	8	3
Low dose (60 mg/month or 120 mg/8 weeks)	1	2
Mid dose (90 mg/month or 120 mg/6 weeks)	2	0
High dose (120 mg/month)	5	1
Oral octreotide (60 mg/day)	0	1

The bolded numbers are for the patient subgroups (medication-naïve, previously-treated, washed out). The unbolded numbers are for the specific acromegaly medications within the previously-treated and washed out subgroups.

Abbreviation: LAR, long-acting release.

^*a*^Most recent acromegaly medication.

### Biochemical Control

**Figure 3.**
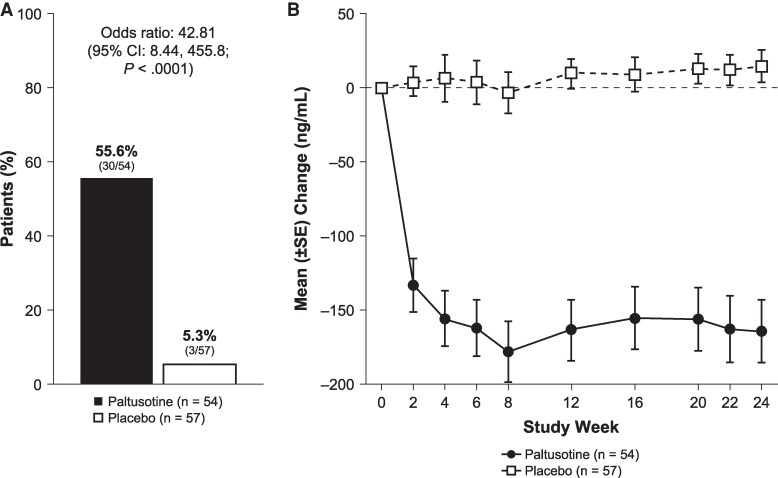
(A) Primary endpoint: proportion of patients with IGF-I ≤ 1.0 × ULN at the end of the randomized controlled phase. The primary endpoint was based on the average of IGF-I values at weeks 22 and 24. If only 1 measurement was available (week 22 or 24), it was used to evaluate the primary endpoint. Patients who discontinued the assigned treatment before week 24 for any reason (including rescue) or had missing IGF-I values at both weeks 22 and 24 were considered not to have met the primary endpoint. Odds ratio is from exact logistic regression models, with treatment group as a factor and stratum as a covariate (for the overall analysis only). (B) Mean change from IGF-I baseline at each assessment. For patients who discontinued randomized treatment before week 24, the last value before rescue (or study discontinuation) was carried forward.

Significantly more patients on paltusotine met the primary endpoint of IGF-I normalization (IGF-I ≤ 1.0 × ULN) at the end of the randomized controlled phase (55.6%; 30/54) compared with the placebo group (5.3%; 3/57) (odds ratio [OR]: 42.81; 95% CI, 8.44-455.82; *P* < .0001; Fig. 3A). Analyses of week 24 completers and the per-protocol population supported the primary analysis (Table S1 (23)). Rescue criteria were met, and rescue medication was administered to 1 patient (1.9%) in the paltusotine group and 13 patients (22.8%) in the placebo group. One additional patient in the paltusotine group also started rescue medication at the investigator's discretion because of the presence of symptoms (the last IGF-I level for this participant before rescue was 1.1 × ULN).

In both stratum 1 (medically untreated at screening) and stratum 2 (washout), a significantly greater proportion of patients treated with paltusotine vs placebo met the primary endpoint: 42.5% vs 2.4% in stratum 1 (OR: 29.21; 95% CI, 4.08-> 999.99; *P* < .0001) and 92.9% vs 13.3% in stratum 2 (OR: 63.04; 95% CI, 5.41-> 999.99; *P* < .0001). Of the patients with previous medical treatment included in stratum 1, 4 of 7 (57.1%) who had not achieved normal IGF-I on prior therapy did achieve biochemical normalization on paltusotine compared with 0 of 6 (0%) treated with placebo. Of those who had achieved control on previous treatment, 6 of 8 (75%) achieved control on paltusotine vs 0 of 6 (0%) with placebo. Of those with unknown prior control, 2 of 3 (66.7%) achieved control with paltusotine vs 0 of 6 (0%) with placebo.

**Figure 4.**
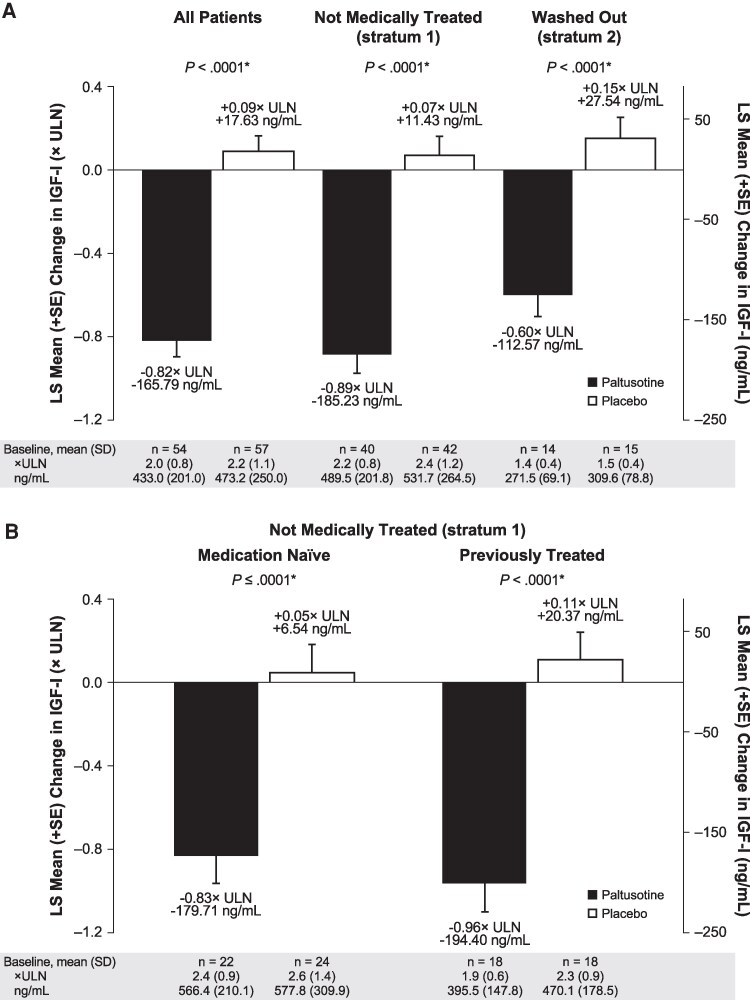
Change in IGF-I at the end of randomized treatment, by acromegaly treatment history. (A) All patients, stratum 1 (not medically treated), and stratum 2 (washed out). (B) Medication-naïve and previously treated patients (from stratum 1). *Comparison of paltusotine vs placebo using analysis of covariance.

**Figure 5.**
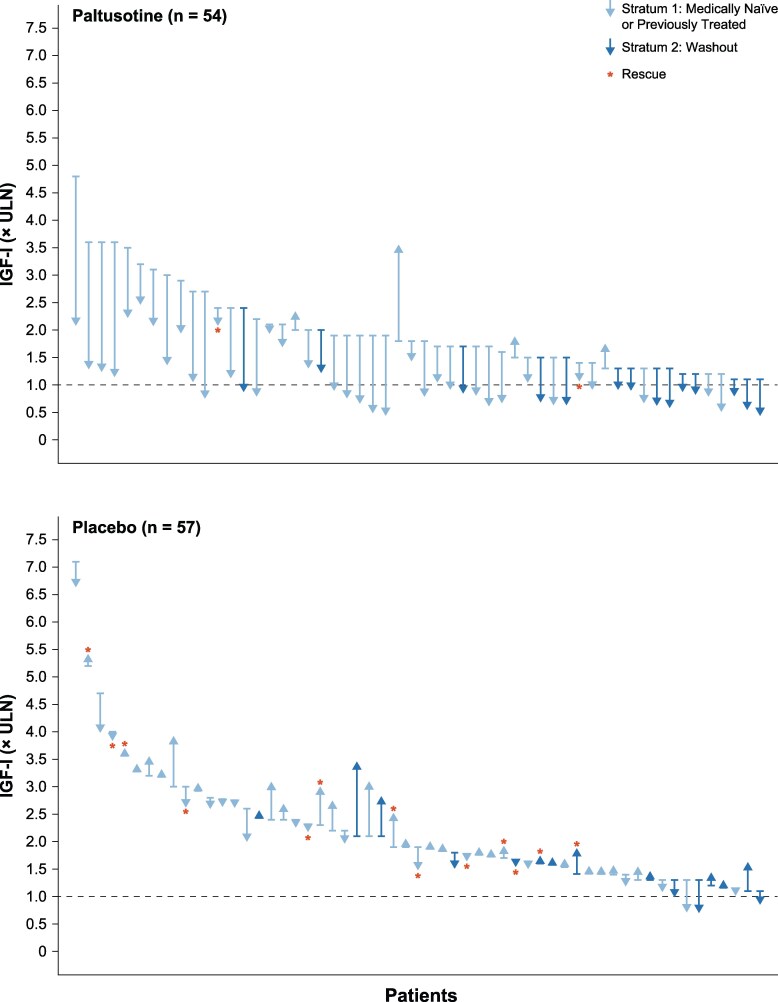
Change in individual IGF-I concentrations from baseline to the end of randomized treatment (average of weeks 22 and 24 or last measurement prior to rescue).

Overall, mean IGF-I was lowered by roughly 40% within 4 weeks of paltusotine treatment initiation and remained suppressed for the duration of the treatment period, whereas mean levels for patients receiving placebo remained largely unchanged from their elevated baseline levels (Fig. 3B). Accordingly, for the key secondary endpoint, the change from baseline IGF-I level at the end of the randomized treatment period for paltusotine-treated patients (-0.82 ± 0.08 × ULN) was significantly greater than in the placebo group, which remained similar to baseline (+0.09 ± 0.08 × ULN), resulting in a treatment difference of -0.91 × ULN (95% CI, -1.11 to -0.71; *P* < .0001). Mean IGF-I was significantly decreased with paltusotine vs placebo in both stratum 1 (medically untreated at screening) and stratum 2 (washed out; Fig. 4A). IGF-I reduction was similar in the medication-naïve and previously treated groups that compose stratum 1 (Fig. 4B). Overall, 92.6% of patients who received paltusotine showed reduced IGF-I levels at the end of the randomized treatment period (Fig. 5).

Median time to first IGF-I ≤ 1.0 × ULN at 2 consecutive visits (regardless of whether IGF-I was in the normal range at the end of randomized treatment) was 44.0 days in the paltusotine group; median time was not reached in the placebo group because few patients experienced IGF-I ≤ 1.0 × ULN (Fig. S1 (23)). The proportion of patients below the IGF-I < 1.3 × ULN threshold at the end of the randomized controlled phase (mean of weeks 22 and 24; secondary endpoint) differed significantly between groups: 66.7% for paltusotine vs 14.0% for placebo (OR: 18.32; 95% CI: 5.64, 79.16; *P* < .0001).

Consistent with the observed changes in IGF-I, mean GH concentration decreased with paltusotine and increased with placebo. Least-squares mean (±SE) change in 5-sample GH at the end of randomized treatment was -2.69 ± 1.9 ng/mL in the paltusotine group and 1.64 ± 1.22 ng/mL in the placebo group (treatment difference: -4.32; 95% CI, -8.54 to -0.11; *P* = .04). The proportion of patients with GH <1.0 ng/mL at week 22 (secondary endpoint) was significantly greater with paltusotine (57.4%) than placebo (17.5%; OR: 7.59; 95% CI, 2.78-23.48; *P* < .0001).

### Determinants of IGF-I Normalization

Baseline IGF-I was the major identified predictor of IGF-I normalization. Although change from baseline IGF-I was similar in all groups evaluated (Fig 4), baseline IGF-I was a key determinant of IGF-I normalization in this study. Baseline IGF-I was 2.5 × ULN in medication-naïve patients, 2.1 × ULN in previously treated patients, and 1.5 × ULN in washed-out patients. Consistent with baseline values, end-of-treatment rates of IGF-I ≤ 1.0 × ULN with paltusotine vs placebo in medication-naïve patients were 22.7% (5/22) vs 4.2% (1/24); in previously treated patients, 66.7% (12/18) vs 0% (0/18); and in washed-out patients, 92.9% (13/14) and 13.3% (2/15).

### Symptom Control

**Figure 6.**
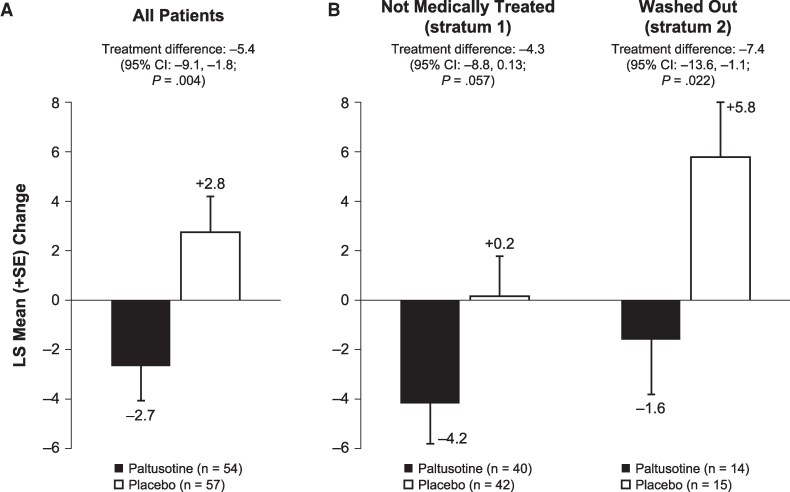
Least-squares mean change from baseline at the end of randomized treatment in Acromegaly Symptom Diary total score (ASD; secondary endpoint) (A) for the overall study population and (B) by stratum. Higher scores indicate worse symptom severity. Treatment difference from worst-rank score analysis of covariance with treatment group as a factor and stratum as a covariate.

**Figure 7.**
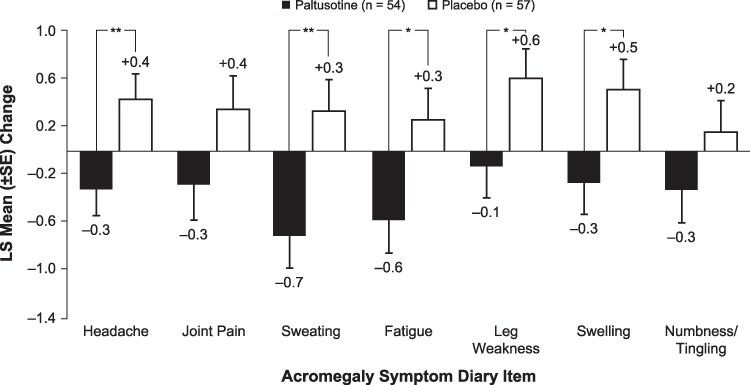
Least-squares mean change from baseline at the end of randomized treatment for Acromegaly Symptom Diary (ASD) item scores in the overall study population. Higher scores indicate worse symptom severity. *P < .05 vs placebo; **P < .01 vs placebo from analysis of covariance with treatment group and stratum as fixed effects and baseline value as a covariate.

The mean completion rate of daily ASD questionnaires, from 14 days before randomization through the end of the randomized controlled phase, was 80.8%. At the end of randomized treatment, the ASD total score (secondary endpoint) was significantly improved in the paltusotine group compared with the placebo group: least-squares mean (±SE) change of -2.7 ± 1.4 vs an increase of 2.8 ± 1.4 (treatment difference: -5.4; 95% CI, -9.1 to -1.8; *P* = .004; Fig. 6A). In patients randomized to placebo, mean ASD score was relatively unchanged in patients who were medically untreated at screening (stratum 1) and continued to worsen in patients who had washed out from SRLs (stratum 2; Fig. 6B). For all core ASD items, mean scores were decreased (ie, improved) with paltusotine and increased (ie, worsened) with placebo; the between-group differences were statistically significant for headache, sweating, fatigue, leg weakness, and swelling (Fig. 7).

### Pituitary Tumor Volume

In patients for whom MRI scans showed visible tumor at baseline, median (range) tumor volume was 441.0 mm^3^ (71-6065) in the paltusotine group (n = 38) and 681 mm^3^ (35-13,658) in the placebo group (n = 49). No patients in either treatment group had a newly visible tumor at the end of randomized treatment. A reduction in tumor volume of >20% was not seen in the placebo group but was observed in 4 patients (11.8%) treated with paltusotine, all of whom had previously undergone pituitary surgery. Three of these patients were naïve to medical treatment at screening and the other was previously treated (≥4 months before screening) with cabergoline. Five patients (13.5%) in the placebo group had a tumor volume increase of >20%: 3 medication-naïve patients with prior pituitary surgery, 1 previously treated patient with prior pituitary surgery, and 1 washout patient with no previous pituitary surgery. No clinically significant increases in tumor volume were observed in the paltusotine group.

### Safety

Overall, 90.7% of patients in the paltusotine group and 86.0% of patients in the placebo group experienced ≥1 AE during the randomized controlled phase (Table 3). The most commonly reported AEs in paltusotine-treated patients were diarrhea, headache, abdominal pain, and arthralgia. Gastrointestinal AEs were rated as mild or moderate and resolved without discontinuing paltusotine. The incidence of AEs classified by the investigator as related to acromegaly was lower for paltusotine (40.7%) than placebo (56.1%). Sinus bradycardia, a known side effect of SRLs, was reported as an AE in 4 (7.4%) paltusotine-treated patients and no patients in the placebo group. Sinus bradycardia was asymptomatic in all cases, and dose interruption was not needed. There were no clinically significant changes in QTcF interval. There was 1 study discontinuation from AEs in each treatment group (paltusotine: mild lipase increase; placebo: severe hepatic enzyme increase). One serious AE was reported: a benign parathyroid tumor that required surgery in a patient in the placebo group.

**Table 3.** Adverse events occurring in ≥5% of patients in either treatment group during the randomized controlled phase

Adverse events, n (%)*^a^*	Paltusotine (n = 54)	Placebo (n = 57)
**Summary of adverse events**		
Any	49 (90.7)	49 (86.0)
Severe*^b^*	2 (3.7)	5 (8.8)
Serious*^c^*	0	1 (1.8)

Common signs and symptoms of acromegaly	Overall	Attributed to acromegaly*^d^*	Overall	Attributed to acromegaly*^d^*
Headache	11 (20.4)	6 (11.1)	19 (33.3)	16 (28.1)
Arthralgia	6 (11.1)	6 (11.1)	13 (22.8)	12 (21.1)
Paresthesia	5 (9.3)	3 (5.6)	3 (5.3)	3 (5.3)
Fatigue	3 (5.6)	2 (3.7)	8 (14.0)	8 (14.0)
Back pain	3 (5.6)	2 (3.7)	4 (7.0)	2 (3.5)
Pain in extremity	3 (5.6)	2 (3.7)	2 (3.5)	1 (1.8)
Asthenia	3 (5.6)	1 (1.9)	3 (5.3)	2 (3.5)
Peripheral swelling	2 (3.7)	2 (3.7)	6 (10.5)	3 (5.3)
Hyperhidrosis	1 (1.9)	1 (1.9)	5 (8.8)	5 (8.8)

Gastrointestinal adverse events	Paltusotine (n = 54)	Placebo (n = 57)
Diarrhea	18 (33.3)	10 (17.5)
Abdominal pain	6 (11.1)	2 (3.5)
Nausea	5 (9.3)	2 (3.5)
Abdominal discomfort	5 (9.3)	1 (1.8)
Constipation	3 (5.6)	1 (1.8)
Dyspepsia	3 (5.6)	6 (10.5)
Abdominal distension	2 (3.7)	3 (5.3)

Other adverse events	Paltusotine (n = 54)	Placebo (n = 57)
Sinus bradycardia	4 (7.4)	0 (0)
Upper respiratory tract infection	4 (7.4)	10 (17.5)
Alopecia	3 (5.6)	1 (1.8)
Furuncle	3 (5.6)	0 (0)
Hyperglycemia	3 (5.6)	0 (0)
Hypertriglyceridemia	3 (5.6)	1 (1.8)
Urinary tract infection	3 (5.6)	4 (7.0)
Cough	2 (3.7)	3 (5.3)
Lipase increased	2 (3.7)	3 (5.3)
Hypertension	1 (1.9)	3 (5.3)
Pyrexia	1 (1.9)	3 (5.3)
Sinusitis	1 (1.9)	4 (7.0)
Insomnia	0	4 (7.0)

Abbreviations: AE, adverse event; MedDRA, Medical Dictionary for Regulatory Activities.

Adverse events were coded using MedDRA version 24.1.

^*a*^Defined as AEs that emerged or worsened during study treatment, including events with onset after rescue medication was administered.

^*b*^Severe AEs were reported in 2 patients in the paltusotine group (1 with headache, 1 with pain in extremity) and 5 patients in the placebo group (2 with headache; 1 with headache and arthralgia; 1 with headache, muscular weakness, and pain in extremity; 1 with hepatic enzyme increase).

^*c*^Serious AE of benign parathyroid adenoma in the placebo group, which was judged by the study investigator as not treatment-related.

^*d*^Classified by the investigator as related to acromegaly.

At baseline, median (interquartile range) hemoglobin A1c was 5.7% (0.6) in the paltusotine group and 5.5% (0.5) in the placebo group. At week 24, median (interquartile range) change from baseline was 0.2% (0.3) and 0.0% (0.3), respectively. Median changes in TSH and free T4 were similar in the 2 treatment groups (Table S2 (23)). Hyperglycemia was reported as an AE in 3 paltusotine-treated patients (5.6%) and no patients in the placebo group. These episodes of hyperglycemia were transient and did not require medication for glucose control. No AEs of hypoglycemia or hypothyroidism were reported.

In patients with no gallstones observed on baseline ultrasound (and no prior cholecystectomy), new gallstones were observed at week 24 in 3 of 34 patients in the paltusotine group and 0 of 37 patients in the placebo group. Cholelithiasis was reported as an AE for 2 patients in each treatment group (paltusotine, 3.7%; placebo, 3.5%).

## Discussion

Paltusotine is the first oral SST2 receptor agonist evaluated for the treatment of patients with biochemically uncontrolled acromegaly not currently receiving medical therapy. This randomized, placebo-controlled trial met all prespecified primary and secondary endpoints, demonstrating that once-daily oral paltusotine can provide both biochemical and symptom control in a wide range of untreated patients with acromegaly and was well tolerated.

The enrolled population in this phase 3 study was modeled after that used in a previous registration trial supporting the approval of injected lanreotide depot (24, 25). Both studies enrolled patients who were naïve to medical therapy at screening and those who stopped an acromegaly therapy, either in the remote (≥4 months before in this trial; >3 months in the previous trial) or recent (washout) past. This population is clinically relevant, reflecting the spectrum of patients with untreated acromegaly that a clinician might encounter in practice.

To be consistent with modern regulatory standards, the primary endpoint of the current trial was prespecified as the proportion of patients with normal IGF-I at the end of the treatment period (26) when using a modern standardized centrally measured assay (20). Although the current assay is likely associated with lower age-normed ULN values than those used in the comparator study (27), the overall rate of IGF-I normalization seen in this trial (55.6%) was similar to that seen in the comparable pivotal trial of lanreotide (54%) (24, 25). Additionally, the rate of IGF-I normalization in the medication-naïve subset after 6 months of treatment with paltusotine in this trial was similar to that seen after 12 months of treatment with injected octreotide long-acting release in a large trial of medication-naïve patients (28). Overall, results suggest that paltusotine is at least as effective as an injected depot SRL for achieving IGF-I normalization in a population of patients with uncontrolled acromegaly. Furthermore, paltusotine treatment was seen to normalize IGF-I in some patients who had failed to achieve this treatment goal or who had an unknown response with other medical therapies in the past.

To provide clinically relevant information complementary to the primary endpoint, change from baseline in IGF-I was prespecified as a key secondary endpoint in this trial and subjected to formal hierarchical statistical testing. IGF-I lowering was shown to be significantly greater with paltusotine than placebo, and relative reduction was not meaningfully different based on treatment history or baseline IGF-I. Decrease in IGF-I was seen in 93% of paltusotine-treated patients and was rapid (typically within the first 4 weeks of treatment), in contrast to injected depot formulations, which require 3 months per dose strength to reach a steady state. Reduced IGF-I was sustained for the duration of the 24-week paltusotine treatment period. Improved biochemical control was also supported by significantly higher rates of GH control, as defined by achieving the guideline target of <1 ng/mL (29) with paltusotine vs placebo.

Along with biochemical indices, the burden of acromegaly symptoms is an important treatment consideration (8, 30). PATHFNDR-1 and PATHFNDR-2 are the first acromegaly trials in which a fit-for-purpose patient-reported outcome instrument was prespecified as a study endpoint, completed on a daily basis, and subject to formal hierarchical statistical testing. The ASD was designed to assess changes in patient-reported symptom severity (22) and was used in 2 previous paltusotine studies (18, 19). In the present study, mean ASD scores were reduced (denoting improvement) in the paltusotine group and increased (indicating worsening) in the placebo group; the overall mean treatment difference was 5.4 points. Symptom change patterns were consistent with expectations for the stratified patient populations. In those patients who were not medically treated recently (stratum 1), symptom scores improved with paltusotine and did not change in patients receiving placebo. In patients who recently stopped depot injections (stratum 2), symptom scores improved slightly with paltusotine treatment, whereas scores of those randomized to placebo substantially worsened.

The impact on residual pituitary tumor size is also an important clinical consideration when evaluating new therapies for acromegaly (31). No increases in tumor volume were observed in paltusotine-treated patients, and there were no newly visible tumors in this study. In the paltusotine group, decreases in tumor volume of >20% were observed in 4 patients, all of whom were medically untreated at screening (stratum 1). In a long-term open-label study, local radiology assessments showed pituitary tumor stability with up to 4 years of paltusotine treatment (32). Data from the OLE phase of this trial will provide additional, longer-term observations of tumor size during treatment with paltusotine.

Most patients with untreated acromegaly who initiate an SRL experience some degree of IGF-I reduction (33). The likelihood of lowering IGF-I sufficiently to reach the normal range is dependent, in part, on the magnitude of baseline elevation (34). Lanreotide was shown to have the highest IGF-I normalization rate in patients who recently washed out of previous therapy, an intermediate rate in patients who discontinued therapy in the remote past, and the lowest rate in patients who were naïve to medical therapy (24). This pattern was also observed in the present study, in which medically naïve patients had the lowest normalization rate in association with the highest baseline IGF-I elevation.

The safety profile in the present study was consistent with that observed in the paltusotine clinical program to date. Common AEs in the paltusotine group included the gastrointestinal AEs commonly observed with SRLs. These adverse effects typically resolved with continued treatment, and no patients discontinued study participation because of gastrointestinal AEs. Other SRL class effects that were more commonly observed with paltusotine relative to placebo were gallstones and sinus bradycardia, indicating that these should be clinical considerations during paltusotine treatment.

In addition to addressing injection-site reactions and other treatment burdens associated with SRL depot injections, strengths of oral paltusotine include the early onset of effect, which allows for more prompt evaluation of biochemical efficacy compared with injected depot SRLs. The ability to titrate more rapidly could facilitate a more streamlined dose optimization process in clinical practice, allow for faster washout to assess the need for ongoing treatment (eg, after radiation therapy), and provide more rapid down-titration or discontinuation when managing the potential adverse effects of SRL therapy. Furthermore, depot injections are associated with cyclic exacerbations of acromegaly symptoms (9), variations in IGF-I levels (14), and frequent inaccurate delivery of drug (10, 12), none of which have been observed with once-daily administration of paltusotine. Both this study and the previous phase 3 randomized controlled trial (PATHFNDR-1) were designed with long-term OLE phases to further evaluate safety parameters, biochemical control, symptom control, and residual pituitary tumor volume during treatment with paltusotine. In the present study, 92.8% of eligible patients elected to enroll in the OLE; a similarly high rate of enrollment (93.0%) was seen for the PATHFNDR-1 OLE (19).

Interpretation of these results is limited by the protocol-specified rescue criteria, which were designed to minimize the time patients were exposed to unacceptable disease control during this double-blind placebo-controlled trial. This required rescue intervention may have resulted in underestimation of true drug-placebo differences in biochemical- or symptom-control parameters.

The study population is representative of the most prevalent untreated, uncontrolled patients with acromegaly in clinical practice. Of note, those who had been on medical therapy in the past were not excluded based on lack of full biochemical control on prior SRL treatment, even though IGF-I normalization was the primary endpoint of the study.

Medications for acromegaly are used based on their ability to lower IGF-I and GH levels and control the symptoms of acromegaly (31). This study presents evidence that these therapeutic goals can be achieved with paltusotine. Biochemical and symptom responses were achieved rapidly and consistently. These responses were sustained for the duration of the treatment period, and paltusotine was well tolerated. Although the previous phase 3 clinical trial demonstrated that paltusotine can maintain biochemical and symptom control in patients switched from injected SRLs, this study showed that paltusotine can achieve control in patients who were not receiving pharmacologic treatment for acromegaly and had elevated IGF-I at the time of randomization. Paltusotine has the prospect of being a once-daily, oral alternative to injected SRLs in patients who require medical therapy, either to maintain control or achieve control of active acromegaly.

## Data Availability

Some or all datasets generated during and/or analyzed during the current study are not publicly available but are available from the corresponding author on reasonable request.

## Clinical Trial Information

ClinicalTrials.gov registration: NCT05192382

## Abbreviations

AE: adverse event
ASD: Acromegaly Symptom Diary
MRI: magnetic resonance imaging
OLE: open-label extension
OR: odds ratio
SRL: somatostatin receptor ligand
SST2: somatostatin receptor 2
ULN: upper limit of normal
